# Irregular and Decayed Teeth

**Published:** 1844-12

**Authors:** 


*Irregular and Decayed Teeth.*—Some times immense mischief arises from the irregular growth of the teeth, and patients have even lost their lives in consequence of it. My friend, Mr. Nasmyth of Edinburg, a most accomplished surgeon and dentist, who took the right way of studying his profession, having been for years a demonstrator of anatomy, met with the following case:—an old friend applied to him with an extensive abscess of the cheek and great swelling of the face and jaw, the abscess extending down to the clavicle. His mouth could not be opened, the inflammation locked the jaw, and the patient ultimately died. On a *postmortem* examination it was found that the cause of the whole mischief was the wisdom-tooth growing forward and lying horizontally instead of perpendicularly. This is a rare case, but it shows you that much mischief and serious consequences may arise from a trifling cause.

From the decay of the teeth you frequently find great mischief arising in the neighborhood. Many cases of ulcerated throat are caused by the presence of stumps, the affection being propagated along the lining membrane. The disease is kept up by, and cannot be removed till the causes are removed. Ulcerations about the lips and nose sometimes arise from disease about the incisors, or are kept up by it. I recollect the case of a gentleman who came from the West Indies to be treated for an ugly ulcerated tumor on the cheek. Very active measures had been adopted in the island where he resided. He had had the part cut out more than once, and corrosive agents applied in all sorts of ways. Recourse had been had more than once to the actual cautery. On the last occasion the surgeon had made him put a lime, a fruit like a small lemon, in the cheek, to distend it, and make the disease more apparent, and then a red hot skewer was thrust into the bottom of the wound. Still it did not heal, and he came here expecting to have some dreadful operation performed for the relief of his malady. The whole case depended on a decayed tooth in the upper jaw: it was taken out, and in a week he was well. He would have recovered at any period of the disease if that tooth had been attended to.—*Liston's Lectures.—Cin. Lancet.*

